# Senescent Cell-Secreted Netrin-1 Modulates Aging-Related Disorders by Recruiting Sympathetic Fibers

**DOI:** 10.3389/fnagi.2020.507140

**Published:** 2020-12-16

**Authors:** Ai Qing Yu, Jie Wang, Xiao Jia Zhou, Ke Yu Chen, You De Cao, Zhi Xiao Wang, Ze Bin Mao

**Affiliations:** ^1^Department of Clinical Laboratory, Hunan Provincial People’s Hospital, the First Affiliated Hospital of Hunan Normal University, Changsha, China; ^2^Hubei Key Laboratory of Embryonic Stem Cell Research, Hubei University of Medicine, Shiyan, China; ^3^Beijing Key Laboratory of Protein Posttranslational Modifications and Cell Function, Peking University Research Center on Aging, Department of Biochemistry and Biophysics, School of Basic Medical Sciences, Peking University Health Science Center, Beijing, China; ^4^Department of Cardiology, Taihe Hospital, Hubei University of Medicine, Shiyan, China

**Keywords:** sympathetic fibers, senescent cells, netrin-1, aged tissues, aging-related disorders

## Abstract

Cellular senescence is implicated in several lines of aging-related disorders. However, the potential molecular mechanisms by which cellular senescence modulates age-related pathologies remain largely unexplored. Herein, we report that the density of sympathetic fibers (SFs) is significantly elevated in naturally aged mouse tissues and human colon adenoma tissues compared to the SFs densities in the corresponding young mouse tissues and human non-lesion colon tissues. A dorsal root ganglion (DRG)-human diploid fibroblast coculture assay revealed that senescent cells promote the outgrowth of SFs, indicating that the senescent cells induce recruitment of SFs *in vitro*. Additionally, subcutaneous transplantation of 2BS fibroblasts in nude mice shows that transplanted senescent 2BS fibroblasts promote SFs infiltration. Intra-articular senolytic molecular injection can reduce SFs density and inhibit SFs infiltration caused by senescent cells in osteoarthritis (OA), suggesting senescent cells promote the infiltration of SFs *in vivo* in aged tissues. Notably, the elevated level of SFs contributes to impaired cognitive function in naturally aged mice, which can be reversed by treatment with propranolol hydrochloride, a non-selective β receptor blocker that inhibits sympathetic nerve activity (SNA) by blocking non-selective β receptors. Additionally, 6-hydroxydopamine (6-OHDA)-induced sympathectomy improved hepatic sympathetic overactivity mediated hepatic steatosis in high fat diet (HFD)-fed *APOE* knockout mice (APOE^−/−^ mice) by reducing hepatic SNA. Taken together, this study concludes that senescent cell-secreted netrin-1 mediated SFs outgrowth and infiltration, which contributes to aging-related disorders, suggesting that clearing senescent cells or inhibiting SNA is a promising therapeutic strategy for improving sympathetic nervous system (SNS) hyperactivity-induced aging-related pathologies.

## Introduction

Cellular senescence is a permanent arrest of cell proliferation in replication-competent but apoptosis-resistant cells (Kirkland and Tchkonia, 2017). This phenomenon was first described by Hayflick (1965) and has become known as the “Hayflick limit,” which is currently defined as replicative senescence. A variety of stimuli and stresses can cause senescence, including telomere shortening, genomic DNA damage, oncogenic insults, metabolic stresses, epigenetic changes, and mitochondrial dysfunction (Kuilman et al., 2010). Despite the different causes of senescence, the senescent cells display several prominent common phenotypes, such as irreversible growth arrest, an enlarged and flat cellular morphology, the elevated activity of senescence-associated-β-galactosidase (SA-β-gal), the formation of senescence-associated heterochromatin foci formation (SAHF) and acquisition of the senescence-associated secretory phenotype (SASP), which includes secreted inflammatory, growth-regulating, and tissue-remodeling factors (Childs et al., 2017; Calcinotto et al., 2019). It has been showed that cellular senescence is causally implicated in biological aging. there is evidence that accumulated senescent cells are implicated in chronologically aging organisms (van Deursen, 2014). Significantly, several lines of evidence have revealed that cellular senescence is closely associated with aging-related diseases, including osteoarthritis (OA), atherosclerosis, cancer, Parkinson’s disease, Alzheimer’s disease, and type 2 diabetes (Bhat et al., 2012; Childs et al., 2016; Calcinotto et al., 2019). The local clearance of senescent cells by senolytic agents attenuates the development of aging-related diseases (Baker et al., 2011; Jeon et al., 2017).

The sympathetic nervous system (SNS) is involved in a multitude of biological phenomena including stress, energy utilization, and physical activity; crucial physical functions that are regulated by the SNS include hemodynamics, temperature regulation, and metabolism (Sorota, 2014). Overactivity of the SNS can result in types of chronic diseases, including cardiovascular disorders and hypertension (Sorota, 2014; de Lucia et al., 2019). Multiple lines of evidence have demonstrated that sympathetic nerve density increases in tumor tissues, those associated with prostate cancer (Magnon et al., 2013), pancreatic cancer (Renz et al., 2018b), liver cancer (Huan et al., 2017), and gastric cancer (Zhao et al., 2014). This promotes tumor development and progression, and denervation of sympathetic nerves or β-adrenergic receptors by surgery suppresses tumorigenesis (Magnon et al., 2013; Zhao et al., 2014; Zahalka et al., 2017). Mechanistically, SNS nerve fiber-secreted norepinephrine promotes never growth factor (NGF) secretion of tumor cells, which in turn recruits SNS never fibers to tumor cells, and ultimately leads to tumorigenesis (Renz et al., 2018b). However, the roles and underlying mechanisms of the SNS in aged tissues and aged-related disorders remains largely unexplored.

Netrin-1, a secreted laminin-related extracellular matrix protein, interacts with the deleted in colorectal cancer receptor (DCC) or the uncoordinated-5 homolog (UNC-5H) receptor to induce chemoattraction or chemorepulsion during axon growth and neuronal migration (Lai Wing Sun et al., 2011). *NTN1*—the gene that encodes netrin-1, acts as oncogene to confer a selective advantage for tumor cell survival by blocking DCC or UNC-5H-induced apoptosis (Fitamant et al., 2008). In normal cells or tissues, netrin-1 participates in regulating angiogenesis, maintaining intestinal homeostasis and regulating epithelial branch morphogenesis, which suggests that netrin-1 is a multifunctional secretory factor (Cirulli and Yebra, 2007; Mehlen et al., 2011). However, the potential role of netrin-1 in senescent cells and aged tissues remains poorly defined.

In the present study, we confirm that senescent cells can induce the infiltration of sympathetic nerve fibers, thus leading to an increase in the density of sympathetic nerve fibers in aging tissues.

Clearing senescent cells by senolytics improved OA and reduced the SF infiltration *in vivo*. Mechanistically, our data showed that senescent cells-induced infiltration of Sympathetic fibers (SFs) was achieved at least partly *via* secreting the axon guidance cue netrin-1. Significance of SFs infiltration in age-related disease is exemplified by our data that brain cognitive decline in naturally aged mice and hepatic steatosis in high fat diet (HFD)-fed mice can be reversed by treatment with propranolol hydrochloride, a non-selective β receptor blocker, and 6-OHDA, a specific Sympathetic nerve toxin respectively. These results suggest that increased sympathetic activity mediated by senescent cells elicited age related disorders, which provides a promising therapeutic strategy for the treatment of aging-related pathologies.

## Materials and Methods

### Cell Lines and Cell Culture

Human 2BS diploid fibroblasts and IMR-90 cells were purchased from the National Institute of Biological Products, Beijing, China. A HEK293 T cell line was preserved by our lab. The cells were cultured in Dulbecco’s modified Eagle’s medium (Invitrogen, USA) supplemented with 10% fetal bovine serum (FBS, Invitrogen, USA), 100 U/ml penicillin and 100 mg/ml streptomycin. All the cell lines were cultured in a humidified incubator at 37°C under 5% CO_2_.

### Animal Care and Ethics Statement

Four-week-old male Balb/c nu/nu mice and 8-week-old male C57BL/6 mice were purchased from the Animal Centre of Peking University Health Science Center. The mice were housed in a temperature- and light-controlled specific pathogen-free (SPF) animal facility with free access to food and water.

The naturally aged male mice were fed on a normal diet for at least 24 months. All experiments involving the handling of mice were approved by the animal ethics committee of Peking University Health Science Centre. The human tissue samples were obtained with informed consent, and the study was approved by the Clinical Research Ethics Committee.

### Dorsal Root Ganglion (DRG) Isolation and Coculture

We carried out DRG isolation according to the protocol described in the literature (Khaminets et al., 2015). DRG–human diploid fibroblast was conducted in accordance with a previously published method (Ceyhan et al., 2008; Wang et al., 2015). Briefly, 2 × 10^5^ cells were suspended in 25 μl of growth-factor-reduced Matrigel (no. 356230, Corning, USA) and placed at the center of a 6 cm petri dish. DRGs were also seeded in 25 μl of Matrigel and placed at exactly 1 mm distance from the cell suspension. Each petri dish was then placed for 20 min in a humidified incubator at 37°C under 5% CO_2_ to allow the Matrigel to polymerize. To enable the formation of a potential signal molecule gradient within the interacting cells and DRGs, a 1 mm-long Matrigel “bridge” was built between the cell suspension and the DRG suspension. After solidification, neurobasal medium (no. 10888022, Invitrogen, USA) supplemented with 10% FBS, 100 U/ml penicillin, 100 μg/ml streptomycin, 0.5 mM L-glutamine and 2% B-27 (no. 17504044, Invitrogen, USA) was added to each petri dish and renewed every 2 days. The petri dishes were photographed under an inverted microscope.

### Analysis of Immunohistochemistry (IHC)

IHC analysis was performed as described previously (Li et al., 2018). Briefly, the formalin-fixed paraffin sections were deparaffinized, rehydrated, and pre-treated with 3% hydrogen peroxide (H_2_O_2_) for 20 min to block endogenous peroxidase. The antibody-binding epitopes of the antigens were retrieved by microwave treatment at 98°C for 10 min, and the sections were then preincubated with 10% goat serum to block nonspecific binding. Rabbit anti- tyrosine hydroxylase (anti-TH; no. ab112; Abcam, USA), rabbit anti-p16 (no. ab189034, abcam, Burlingame, CA, USA), rabbit anti-netrin-1 (no. GTX37361, GeneTex, USA), and rabbit anti-EZH2 (no. 5642s, CST, USA), diluted a at ratios of 1:100, 1:200, 1:50, 1:100 respectively, were used as the primary antibodies The specimens were incubated overnight with the primary antibodies 4°C, followed by the addition of biotinylated anti-rabbit or anti-mouse secondary antibodies and streptavidin-horseradish peroxidase. We used 3,3′-diaminobenzidine (DAB) as a chromogen, and hematoxylin and eosin (HE) for counterstaining.

### Immunoblots and Antibodies

The whole cell lysates was prepared by incubating the cells in lysis buffer containing protease inhibitor cocktail (no. 05892791001, Roche, Switzerland) and a phosphatase inhibitor (P1092, Beyotime Biotechnology, China). The supernatants were separated by sodium dodecyl sulfate–polyacrylamide gel electrophoresis (SDS–PAGE; 10% sodium dodecyl sulfate), then electrotransferred onto a polyvinylidene difluoride (PVDF) membrane (Bio-Rad, USA). After blocking with 5% skimmed milk powder, we incubated the membranes overnight with primary antibodies against P16 (no. ab189034; 1:1,000 dilution; Abcam, Burlingame, CA, USA), EZH2 (no. 5642s; 1:1,000 dilution; CST, USA) and β-actin (no. PM053; 1:1,000 dilution; MBL, Japan) at 4°C overnight. After washing them three times, we then incubated the membranes with secondary antibodies (1:10,000 dilution; EarthOx Life Sciences, USA) at room temperature for 1 h. Finally, we used an enhanced chemiluminescence (ECL, Millipore, USA) kit for visualization.

### Netrin-1 Enzyme-Linked Immunosorbent Assays (ELISAs)

Netrin-1 ELISAs assay was carried out to determine the level of netrin-1 in the conditioned media from young and senescent cells using a Human Netrin-1 ELISAs Kit (no. E1827h; Elabscience, China) according to the manufacturer’s instructions.

### RT-qPCR

Total RNA was extracted using Trizol reagent (no.15596018; life technology, USA). Complementary DNA (cDNA) was produced using a ReverTra Ace^®^qPCR RT kit (no. FSQ101, TOYOBO, Japan) according to the manufacturer’s instructions. The cDNA samples were amplified using a SYBR^®^ Green Realtime PCR Master Mix (no. QPK201, TOYOBO, Japan) in 7500 Real-Time PCR System (Applied Biosystems). The relative expression was normalized to the expression level of GAPDH (the gene that encodes glyceraldehyde 3-phosphate dehydrogenase; GAPDH). The primer sequences are provided in Table 1.

**Table 1:** Primers sequences used for quantitative reverse transcription polymerase chain reaction (RT-qPCR).

Oligonucleotides sequences (F: Forward, R: Reverse) for Realtime PCR	5′→3′
CXCL1-F (Human)	TCGAAAAGATGCTGAACAGTG
CXCL1-R (Human)	AGCGATGCTCAAACACATTAG
CXCL2-F (Human)	CAATGCCCCAGTCACCTGCTG
CXCL2-R (Human)	ACAGATCTCCTTGGCCACAATG
IL1α-F (Human)	AGATGCCTGAGATACCCAAAACC
IL1α-R (Human)	CCAAGCACACCCAGTAGTCT
IL1β-F (Human)	ATGATGGCTTATTACAGTGGCAA
IL1β-R (Human)	GTCGGAGATTCGTAGCTGGA
IL-6-F (Human)	CCTGAACCTTCCAAAGATGGC
IL-6-R (Human)	TTCACCAGGCAAGTCTCCTCA
CXCL-8-F (Human)	ACTGAGAGTGATTGAGAGTGGAC
CXCL-8-R (Human)	AACCCTCTGCACCCAGTTTTC
NGF-F (Human)	TGTGGGTTGGGGATAAGACCA
NGF-R (Human)	GCTGTCAACGGGATTTGGGT
BDNF-F (Human)	GGCTTGACATCATTGGCTGAC
BDNF-R (Human)	CATTGGGCCGAACTTTCTGGT
NTF3-F (Human)	CCCGAGAGCCGGAGCGGGGA
NTF3-R(Human)	GTGACTCTTATGCTCCGCGT
NTF4-F(Human)	AGCGAAACTGCACCAGCGAG
NTF4-R (Human)	CACCTTCCTCAGCGTTATCA
NTN1-F (Human)	GCAAGAGCGGAGGTGTCTGCCT
NTN1-R (Human)	GCTTGCCCATGTCGCGGTAGT
GAPDH-F (Human)	ACAACAGCCTCAAGATCATCAGCAAT
GAPDH-R (Human)	GTCCTTCCACGATACCAAAGTTGTCA

### Chromatin Immunoprecipitation (ChIP) Assay

ChIP assay was conducted using a ChIP Assay Kit (no. 17-295; Millipore, USA) according to the manufacturer’s instructions. Briefly, we crosslinked 4 × 10^7^ cells with 37% formaldehyde– phosphate-buffered saline (PBS) for 10 min at 37°C. After washing them twice with ice-cold PBS, we scraped the cells into conical tubes, and resuspended them using a lysis buffer. We then sonicated the cells to shear the DNA into lengths of between 200 and 1,000 base pairs and centrifuged them to remove the insoluble material. The supernatants were collected and incubated overnight at 4°C with the indicated antibodies and protein G magnetic beads. The beads were washed, and the precipitated chromatin complexes were collected, purified, reverse-crosslinked at 65°C for 4 h, and incubated at 95°C for 10 min. We purified the precipitated DNA fragments using the QIAquick PCR purification kit (no. 28106, Qiagen, German), and then quantified them by quantitative reverse transcription polymerase chain reaction (RT-qPCR) analysis with the indicated primer pair.

### Immunofluorescence Staining

Briefly, the cells were fixed with 4% paraformaldehyde at room temperature for 15 min, then treated with 0.5% Triton X-100 to change their permeability. After washing three times with PBS, the cells were blocked with 10% goat serum at room temperature for 30 min, then incubated overnight with anti-MAP-2 (no. ab32454) and β-Tubulin (no. ab18207; dilution 1:100; Abcam, USA) at 4°C overnight. Then the cells were incubated with secondary antibody (no. BA1032; 1:100 dilution; BOSTER, China) for 1 h, and used 4′,6-diamidino-2-phenylindole (DAPI) to counterstain nuclei. Finally, the cells were incubated with chromogen for 5 min and examined under an inverted microscope.

### Senescence-Associated Beta-Galactosidase (SA-β-Gal) Staining

SA-β-gal activity assay was carried out as described previously (Zhuo et al., 2010).

### The Object–Place Recognition

An object–place recognition test was performed according to previously published methods (Zheng et al., 2017; Raihan et al., 2019). Briefly, the male mice were allowed to adapt to a 20 × 20 × 20 cm box marked with visual cues for 30 min per day for 2 days before the test. The test consisted of two phases. In the sample phase, the mice were placed in the box with two identical objects (two cubes, arbitrarily named as *O*A and *OB*) at two different corners. The mice were allowed to explore freely for 10 min, and were then removed from the box for another 10 min, during which time the box and objects were cleaned with by 75% ethanol. Pseudorandomly, one of the two objects (*O*A) was left unchanged and in its original place, and the other (*O*B) was moved to a new corner. For the test phase, the mice were placed into the box again and allowed to explore for 10 min. An exploration was counted when the mouse headed towards or contacted the object with its nose. The bias score was calculated as the exploring time (*O*B − *O*A)/(*O*B + *O*A). Propranolol hydrochloride was dissolved in saline to form a final concentration of 1 mg/ml. Then, prior to the Object–place recognition test, the aged and young mice were intraperitoneally injected with propranolol hydrochloride or saline (20 mg kg^−1^), respectively.

### Osteoarthritis Surgery and Administration of Senolytic Agent

Surgery-induced OA models were established by transecting the medial collateral ligament and removing the medial meniscus according to the method described previously (Zheng et al., 2019). One week after surgery, we performed discontinuous intraarticular injection of senolytic dasatinib (5 mg kg^−1^) and quercetin (6 mg kg^−1^) at days 7, 14, 21, and 28 unilaterallly into the left-knee joint cavity once weekly (Roos et al., 2016). After intraarticular injections, the whole joints were taken out for the subsequent experiments.

### Hepatic Sympathetic Nerve Denervation by 6-Hydroxydopamine

After 5 weeks of HFD or normal chow feeding, male mice were randomly assigned to either liver denervation or sham surgery. Intra-peritoneal injection of 6-hydroxydopamine (6-OHDA; 150 mg kg^ −1^) or vehicle control (saline) was performed in normal chow and HFD fed mice (Hurr et al., 2019). 6-OHDA is a well-documented neurotoxin that results in the destruction of catecholaminergic (i.e., sympathetic) nerves (Magnon et al., 2013). After 3 days of 6-OHDA injection, mice were euthanized and the liver tissues were taken out for the subsequent experiments.

### The Quantitative Analysis

The quantitative analysis for IHC is conducted by imageJ software by analyzing the optical density intensity and for coculture is conducted by photoshop software by analyzing the immigration distance of SFs from DRG.

### Statistical Analysis

The quantitative data from three independent biological repeats was used as the means ± standard deviations (SDs). The statistical analyses were performed using 25.0 SPSS software, and statistical significance between groups was determined by Student’s *t*-tests or analysis of variance (ANOVA). A value of *P* < 0.05 indicates that the difference is statistically significant. *P < 0.05, **P < 0.01, ***P < 0.001. N.S. denotes no significance.

## Results

### SFs Density Level Is Markedly Elevated in Aged Tissues

**Figure 1:**
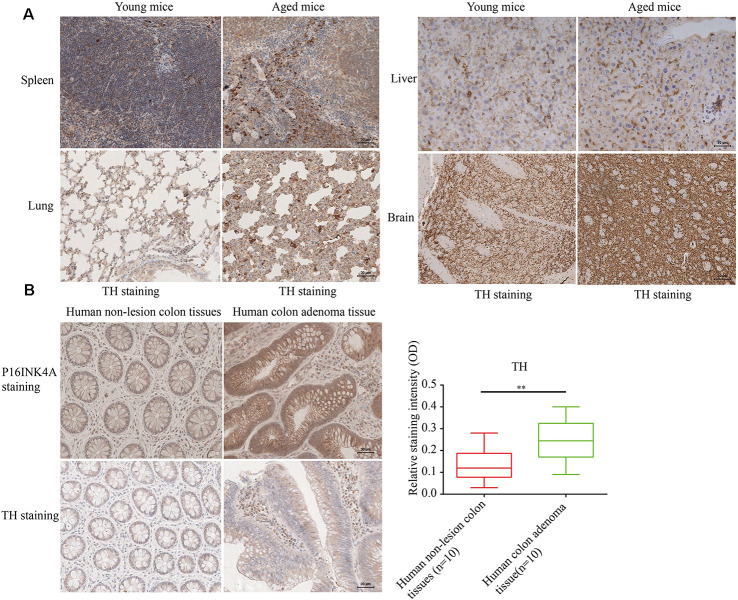
Elevated sympathetic fiber (SFs) density in aged tissues. **(A,B)** SFs were identified by immunohistochemistry (IHC) with anti-tyrosine hydroxylase (TH) antibodies in brain, liver, spleen tissue and lung tissue samples from naturally aged mice (*n* = 3), and human colon adenoma tissue samples (*n* = 10), **P < 0.01, bars, mean ± SD, scale bar, 30 μm. The cerebral hemisphere parenchyma in brain was employed for IHC experiments.

To determine the relation of cellular senescence to outgrowth of sympathetic nerves, The SFs density was detected [identified by tyrosine hydroxylase (TH) staining] in naturally aged male mice (24-month-old) and human colon adenoma tissues, two widely accepted aging models (Jin et al., 2014; Farr et al., 2017; identified by p16^INK4A^ staining and SA-gal staining). As shown in Figure 1A (showing a representative example of one of the pairs of samples), aged mice displayed an elevated density of SFs in examined tissues compared to young mice. Furthermore, elevated SFs were also observed in human colon adenoma tissue, a precancerous lesion which is widely accepted in aging models (Figure 1B). The p16^INK4A^ and SA-gal staining of the 24-month-old and 8-month-old mouse tissues are provided in Supplementary Figure 1. This suggests that cellular senescence is associated with the infiltration of SFs.

### Senescent Cells Promote Sympathetic Nerve Outgrowth Both *In Vitro* and *In Vivo*

**Figure 2:**
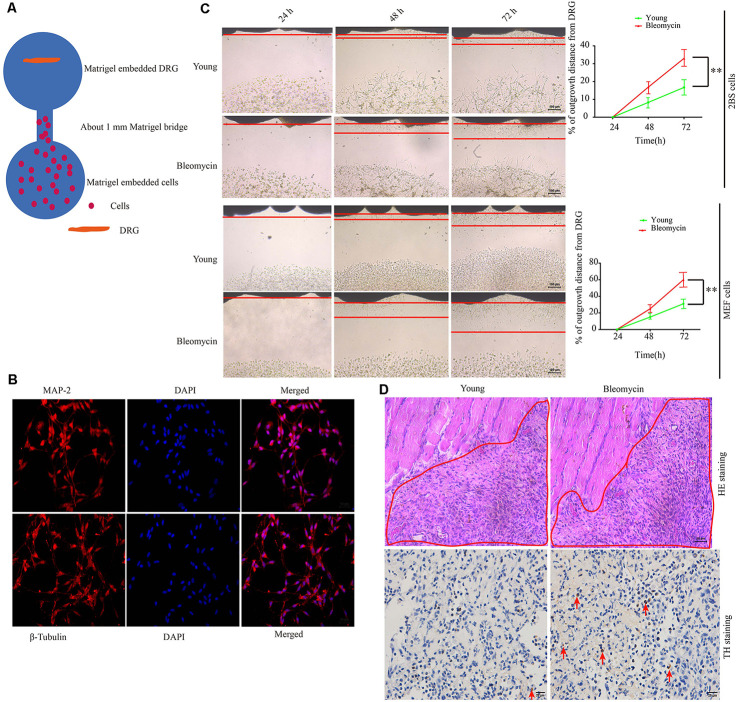
Senescent cells contribute to increased SFs densities, both *in vitro* and *in vivo*. **(A)** Schematic illustration of the coculture model. **(B)** Identification of dorsal root ganglion (DRG)-derived neurons by immunofluorescence with MAP-2 and β-tubulin antibody scale bar, 10 μm. **(C)** DRG cocultures with young human diploid fibroblasts (2BS), 2BS fibroblasts with bleomycin-induced senescence, young mouse embryonic fibroblasts (MEFs), or MEFs with bleomycin-induced senescence, **P < 0.01, scale bar, 100 μm. **(D)** Upper panel: hematoxylin and eosin (HE) staining of the subcutaneous tissue transplanted with treated 2BS; the red box indicates the transplant site of the 2BS fibroblasts, scale bar, 50 μm, *n* = 3. Lower panel: tyrosine hydroxylase (TH) IHC staining of the subcutaneous tissue at the transplant site of the 2BS fibroblasts to identify SFs scale bar, 30 μm, *n* = 3.

**Figure 3:**
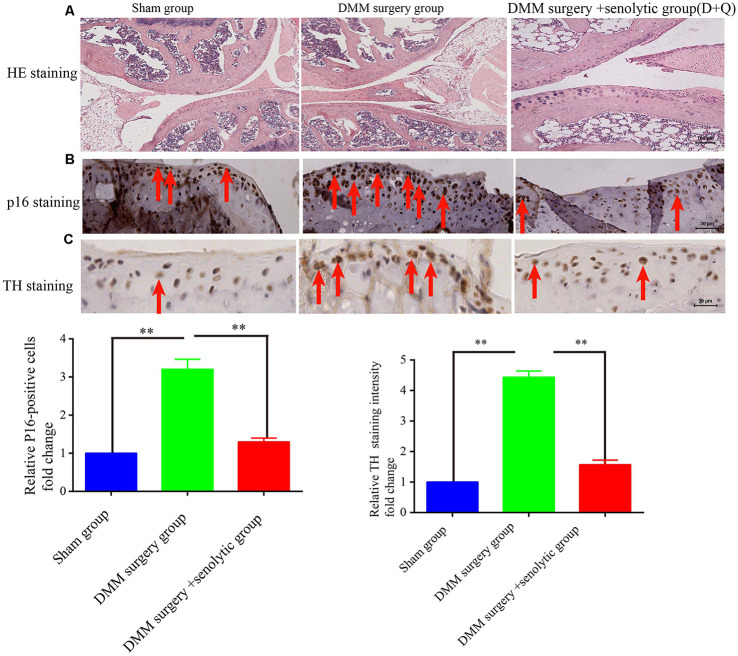
Clearing senescent cells by senolytic decreases the SFs infiltration in mouse OA model. **(A)** Hematoxylin and eosin (HE) staining of joint from sham control, OA, and OA+ senolytics (D + Q) group scale bar, 100 μm. **(B)** IHC for P16^INK4A^ staining in articular cartilage of joint from sham control, OA, and OA+ senolytics (D + Q) group. Red arrows indicate positive P16^INK4A^ staining, **P < 0.01 scale bar, 30 μm. **(C)** TH staining in articular cartilage of joint from sham control group, OA group and OA+ senolytics (D + Q) group, Red arrows indicate positive TH staining, bars, mean ± SD, **P < 0.01, scale bar, 30 μm, *n* = 3.

At first, we hypothesized that the increased SF density results from senescent cells in aged tissues. To validate this hypothesis, three experiments were conducted as below. First, human young 2BS fibroblasts [population doublings (PD) 23] were induced to senescence by different stimuli which include bleomycin (a DNA damaging agents), p16 over-expression and Serially passage, and then *in vitro* coculture models were established using these senescent cells and DRGs (DRG) isolated from 8-week-old Sprague Dawley (SD) rats (Figure 2A). A DRG-derived neuron was identified by neuron specific markers MAP-2 and β-tubulin with immunofluorescence staining (Figure 2B). Results showed that all three types of senescent cells could evidently promote SF outgrowth after 72 h coculture compared to corresponding control groups (Figure 2C, Supplementary Figures 2A–E). The same phenomenon was also observed in senescent mouse embryonic fibroblasts (MEF) which was induced by bleomycin (Figure 2C). This suggests that senescent cells promote SFs outgrowth *in vitro* with a senescent induction and cells type-independent manner. Second, 1 × 10^7^ young fibroblasts (PD 20) or 1 × 10^7^ bleomycin-induced senescent fibroblasts were subcutaneously xenografted into male BALB/c nu/nu mice. Seven days after transplantation, the subcutaneous tissue at the transplant site were removed for IHC staining with TH antibody. The IHC assays revealed that the SFs were recruited to the subcutaneous area bearing the xenografted senescent cells but not young cells (Figure 2D). Our data show that senescent cells facilitate the SF outgrowth toward senescent cells *in vivo*. We next investigate whether clearing senescent cells *in vivo* could abrogate the SFs infiltration. OA, which is frequently used as an aging model, was induced by a transection of the medial collateral ligament and destabilization of the medial meniscus (DMM) surgery (Kim et al., 2014). Results showed that the OA group exhibited increased p16^INK4A^ expression and SFs density compared with the sham group, which is consistent with previously published articles. To observe the relation of senescent cells to SFs elevation, Dasatinib (D) + Quercetin (Q), a senolytic molecule that selectively killed senescent cells in DMM surgery-induced OA, was intra-articularly injected. D + Q was injected immediately after DMM surgery to prevent the recruitment of sympathetic nerves by early senescent cells during the experiment. As showed in Figures 3A–C, selective removal of the senescent cells reduces SFs density of OA. This suggests that senescent cells might play a causative role in elevated SF density of OA. Therefore, senescent cells accelerate the SF outgrowth both *in vitro* and *in vivo*.

### Senescent Cells Recruit SFs *via* Secreting Axon Guidance Cues Netrin-1

**Figure 4:**
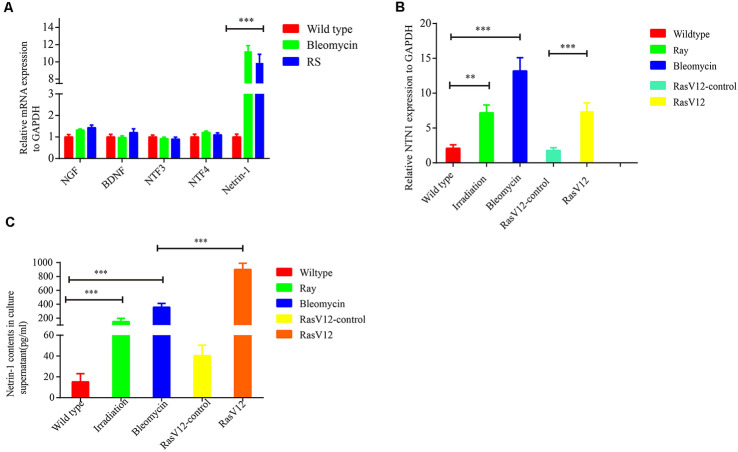
Senescent cells-secreted netrin-1 facilitates SFs outgrowth *in vitro*. **(A)** Quantitative reverse transcription polymerase chain reaction (RT-qPCR) determination of the relative expression levels of never growth factor (NGF), brain-derived neurotrophic factor (BDNF), NTF3, NTF4 and NTN1 in young 2BS fibroblasts, 2BS fibroblasts with bleomycin-induced senescence, or replicative senescent 2BS fibroblasts. **(B,C)** Detection of NTN1 mRNA levels and protein levels in 2BS fibroblasts with irradiation-induced senescence and bleomycin-induced senescence, RasV12-induced senescence and in the corresponding controls, respectively. **P < 0.01, ***P < 0.001, bars, mean ± SD.

To explore the mechanism underlying senescent cells recruiting SFs, the expressions of several neurotrophins (NTs) and axonal guidance cues were examined, which include nerve growth factor (NGF), brain-derived neurotrophic factor (BDNF), NT3, NT4 and netrins, in senescent cells. The data from qRT-PCR revealed that netrin-1 was dramatically upregulated in the bleomycin-induced premature senescent cells or in the replicative senescent cells, while the expression levels of NGF, BDNF, NT3, and NT4 were not significantly altered (Figure 4A). Additionally, Netrin-1 expression was also markedly upregulated in premature senescent 2BS fibroblasts induced by bleomycin, 10GY-irradiation- or RasV-12 at the transcriptional levels (Figure 4B). ELISA further confirmed the elevated expression of netrin-1 at protein level in the abovementioned senescent cells (Figure 4C). Furthermore, it was found that the netrin-1 protein level also increased dramatically in naturally aging mice tissues and human colon adenoma tissues in comparison to young mice tissues and human non-lesion colon tissues respectively (Supplementary Figure 3).

**Figure 5:**
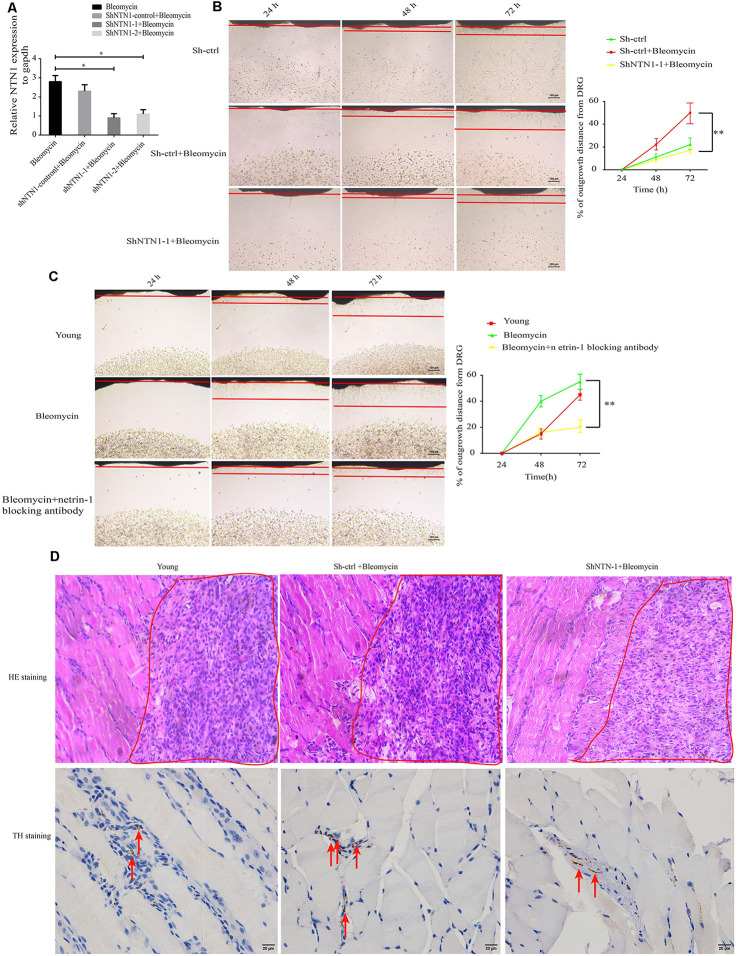
Knockdown or blockage of netrin-1 impedes SFs outgrowth, both *in vitro* and *in vivo*. **(A)** Quantitative reverse transcription polymerase chain reaction (RT-qPCR) analysis was used to determine the efficiency of *NTN1* knockdown by a short hairpin RNA (shRNA), *P < 0.05. **(B)** DRG cocultures with the following: short hairpin control-treated 1 × 10^7^ 2BS fibroblasts (sh-ctrl 2BS); 1 × 10^7^ 2BS fibroblasts with bleomycin-induced premature senescence; and 1 × 10^7^ 2BS fibroblasts with bleomycin-induced premature senescence that had been treated with short hairpin RNA targeting NTN1 (shNTN1 2BS+ bleomycin), **P < 0.01, scale bar, 100 μm. **(C)** DRG cocultures with the following: young mouse embryonic fibroblasts (MEFs); MEFs with bleomycin-induced senescence; and netrin-1 blocked MEFs with bleomycin-induced senescence, **P < 0.01, scale bar, 100 μm. **(D)** Upper panel: hematoxylin and eosin (HE) staining of the subcutaneous tissue transplanted with treated 1 × 10^7^ young 2BS fibroblasts, 1 × 10^7^ sh-ctrl 2BS fibroblasts with bleomycin-induced premature senescence, and 1 × 10^7^ sh-NTN-1 2BS fibroblasts with bleomycin-induced premature senescence; the red box indicating the eosin (HE) staining of the transplant site of the indicated 2BS fibroblasts, scale bar, 50 μm, *n* = 3. Lower panel: IHC of tyrosine hydroxylase (TH) of the subcutaneous tissue at the site at which the indicated 2BS fibroblasts were transplanted for the identification of SFs, scale bar, 20 μm, *n* = 3.

To verify the effect of elevated netrin-1 in senescent cells on attracting SFs, *NTN1* was knocked down using 3′-untranslated region (3′-UTR) targeting short-hairpin RNA (shRNA) in the 2BS fibroblasts, and determined the NTN1 knockdown efficiency by qRT-PCR (Figure 5A). then DRGs were cocultured with each of the following: 2BS fibroblasts treated with a short hairpin RNA control (sh-ctrl 2BS); 2BS fibroblasts with bleomycin-induced premature senescence; and NTN-1-knocked-down 2BS fibroblasts (sh-NTN-1 2BS) with bleomycin-induced premature senescence. The coculture results uncovered that netrin-1 knockdown 2BS fibroblasts with bleomycin-induced premature senescence abolished senescent 2BS-induced SFs outgrowth compared to 2BS fibroblasts with bleomycin-induced premature senescence (Figure 5B). Furthermore, the using of a netrin-1 blocking antibody also eliminated SFs outgrowth induced by MEF fibroblasts with bleomycin-induced premature senescence (Figure 5C). Our data suggest that senescent cell -secreted netrin-1 promotes SFs outgrowth *in vitro*.

We further reasoned that senescent cells can promote SFs outgrowth *in vivo*. Nest, 1 × 10^7^ young 2BS fibroblasts, 1 × 10^7^ sh-ctrl 2BS fibroblasts with bleomycin-induced premature senescence, and 1 × 10^7^ sh-NTN-1 2BS fibroblasts with bleomycin-induced premature senescence were subcutaneously xenografted into male BALB/c nu/nu male mice. Seven days after transplantation, these tissues from the transplant site were removed for IHC assays with anti-TH antibody. The IHC assays revealed that netrin-1 knockdown eliminates senescent 2BS-induced SFs outgrowth *in vivo* (Figure 5D).

### Netrin-1 Expression Is Negatively Regulated by EZH2 in Human Diploid Fibroblasts

**Figure 6:**
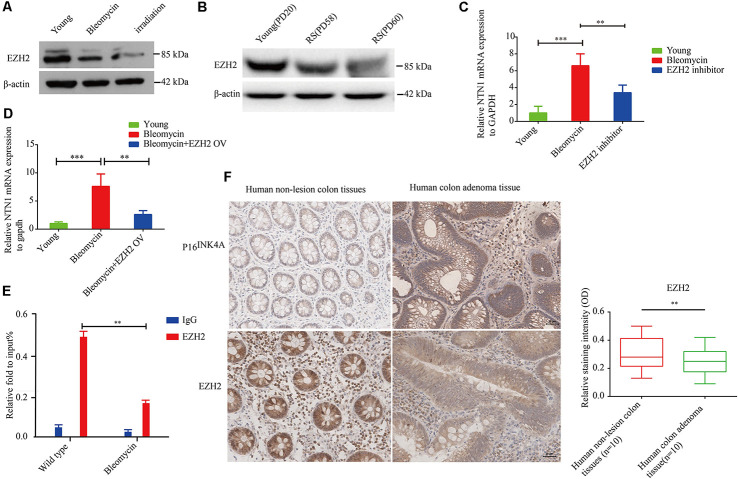
EZH2 negatively regulates netrin-1 expression in human diploid fibroblasts. **(A)** Immunoblot analysis detected EZH2 protein expression in young 2BS fibroblasts, and 2BS fibroblasts with bleomycin- or-irradiation-induced senescence. **(B)** Immunoblot analysis detected EZH2 protein expression in young 2BS fibroblasts and replicate senescent 2BS fibroblasts. **(C)** Quantitative reverse transcription polymerase chain reaction (RT-qPCR) analysis was used to determine the level of NTN1 mRNA in young 2BS fibroblasts, bleomycin-induced senescent 2BS fibroblasts, young 2BS fibroblasts treated with EZH2 inhibitor. **(D)** RT-qPCR detected the level of NTN1 mRNA in young 2BS fibroblasts, bleomycin-induced senescent 2BS fibroblasts and bleomycin-induced senescent 2BS fibroblasts with EZH2 overexpression. **(E)** Chromatin immunoprecipitation (ChIP) analysis of the binding of EZH2 on the NTN promoters in young 2BS fibroblasts and 2BS fibroblasts with bleomycin-induced senescence. **(F)** EZH2 expression in human colon adenoma tissues and non-lesion tissues. **P < 0.01, ***P < 0.001, bars, mean ± SD, scale bar, 30 μm, *n* = 10.

Enhancer of zeste homolog 2 (EZH2) is a component of polycomb repressive complex 2 (PRC2) with an enzymatic subunit that catalyzes the methylation in Lys27 of histone H3 (H3K27), thereby repressing EZH2 represses the transcription of coding genes (Sermer et al., 2019). In the present study, we found that EZH2 expression was significantly down-regulated in premature senescent 2BS fibroblasts and 10GY -irradiation- or bleomycin-induced premature senescence in the corresponding comparison to young 2BS fibroblasts (Figures 6A,B). Moreover, netrin-1 mRNA levels in EZH2 inhibitor-treated young 2BS fibroblasts increased dramatically compared to the netrin-1 mRNA levels in non-treated young 2BS fibroblasts (Figure 6C). EZH2 overexpression could reverse the enhanced netrin-1 mRNA levels in bleomycin-induced premature senescence 2BS fibroblasts (Figure 6D). Furthermore, the Chip assay revealed that the percentage of EZH2 enrichment on the Ntn1 promoters in the young 2BS was significantly greater than in the 2BS fibroblasts with bleomycin-induced premature senescence (Figure 6E), suggesting that senescent 2BS upregulated Netrin-1 expression by reducing EZH2 binding to the NTN1 promoter. We also found that the EZH2 expression levels were reduced in the human colon adenoma tissue samples compared to those in the normal colon tissue samples (Figure 6F).

### Inhibition of Sympathetic Nerve Activity Alleviates Age-Related Disorders

**Figure 7:**
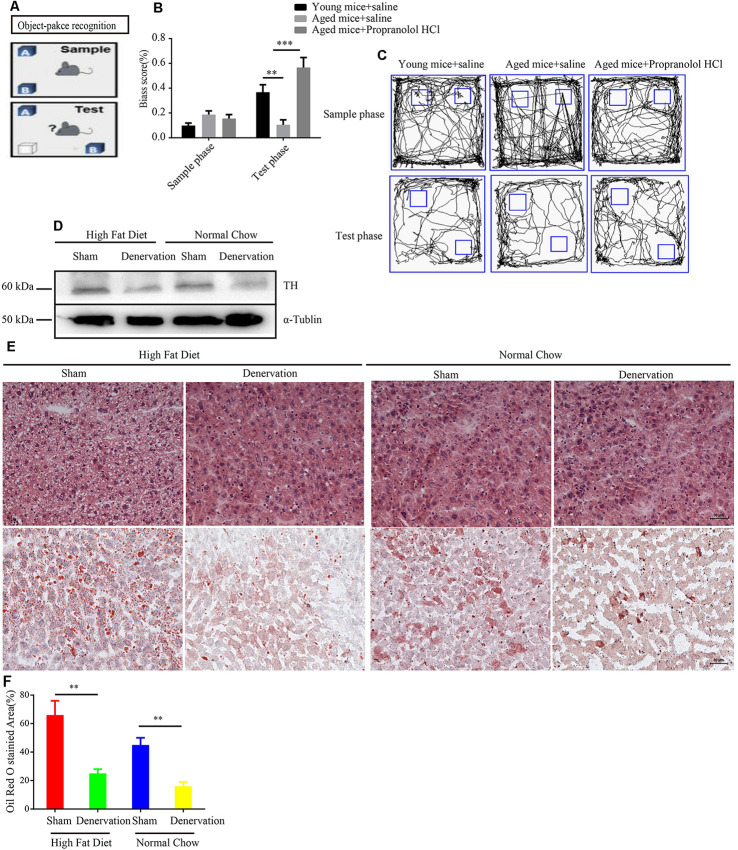
Inhibition of sympathetic nerve activity (SNA) alleviates age-related disorders. **(A)** The object–place recognition task. One object (*O*_B_) was moved to a novel place in the test phase, and the bias score was calculated as exploring times (*O*_B_−*O*_A_)/(*O*_B_+ *O*_A_) in sample and test phases. **(B)** Weakened object–place recognition in naturally aged mice was reversed by treatment with propranolol hydrochloride, **P < 0.01, ***P < 0.001, bars, mean ± SD (*n* = 12 for each group). **(C)** Representative examples of the orbits followed by the mice when exploring object (O_A_) and object (O_B_) in the sample and test phases. **(D)** Western blot detected the expression level of tyrosine hydroxylase (TH) in high fat diet (HFD) and normal chow APOE^−/−^ mice treated with sham (saline) or denervation (6-OHDA). **(E)** Hematoxylin and eosin (HE) staining and Oil Red O staining of liver tissues from HFD and normal chow APOE^−/−^ mice treated with sham (saline) or denervation (6-OHDA), scale bar, 30 μm. **(F)** Statistical analysis of Oil Red O stained area of liver tissues from HFD and normal chow APOE^−/−^ mice treated with sham (saline) or denervation (6-OHDA), **P < 0.01, bars, mean ± SD (*n* = 3).

Elevated sympathetic nerve activity (SNA) has been demonstrated in patients with several chronic diseases. To assess the functional role of sympathetic nerve in aging-related disease. Propranolol hydrochloride was intraperitoneally injected into 24-years-old male mice to observe the effect of inhibiting SNA on the brain function, as the above experiment reveals that SFs density is significantly increased in the brain tissue of naturally aged mice. An object–place recognition experiment was conducted to evaluate the cognitive performance of mice (Figure 7A). As shown, aging mice exhibited an impaired cognitive performance compared to young mice, which is manifested in reduced bias scores toward objects removed to novel places in the test phase. Importantly, propranolol hydrochloride treatment may significantly improve object–place cognitive ability in aging mice, suggesting that elevated SNA in brain tissues impairs cognitive performance (Figures 7B,C). This view is supported by a meta-analysis of individual participant data from prospective cohort studies, which reveals that antihypertensive medication including β receptor blocker lowered the risk for dementia and Alzheimer’s disease. We also performed intra-peritoneal injection of 6-OHDA into normal chow and HFD fed APOE^−/−^ mice to denervate hepatic sympathetic nerve (Figure 7D). We found 5 weeks HFD induced hepatic steatosis compared to normal chow in APOE^−/−^ mice. However, 6-OHDA-induced hepatic denervation reduces HDF-mediated hepatic steatosis compared to sham-treated HFD APOE^−/−^ mice (Figures 7E,F).

Collectively, these data point to the conclusion that elevated SNA is involved in age-related disease.

## Discussion

The SNS is involved in a multitude of biological phenomena including stress, energy utilization, and physical activity—crucial physical functions. SNS deregulation can result in types of chronic diseases, including cardiovascular disorders and cancer. Sympathetic nerve overactivity is increasingly recognized as a hallmark feature linking aging and obesity with increased cardiovascular risk. Recently, Hurr et al. (2019) reported that hepatic SNA elevates in a murine model of HFD-induced hepatic steatosis which is associated with hepatocytes senescence. However, the mechanism underlying sympathetic nerve overactivity in age-related disorder is unclear. In present study, we demonstrate that senescent cells accumulation is an important factor of elevated SF density and SNA in age tissues. Of course, the influence of other factors on SNA cannot be ruled out.

There is a growing body of evidences that the accumulation of senescent cells in aging tissues promotes aging-related pathologies; myriad studies have proven that clearing local accumulated senescent cells ameliorates aging-related diseases such as OA and atherosclerotic (Roos et al., 2016; Farr et al., 2017). At present, most publications attest that a senescent cell-generated SASP contributes to aging-related pathologies in an autocrine or paracrine fashion. For instance, in OA, senescent chondrocytes secrete metalloproteinase that degrade cartilage in atherosclerosis (Billinghurst et al., 1997), senescent foam cells secrete CCL2 and VCAM1 to recruit monocytes and trigger their conversion into senescent foam cells and in intervertebral degeneration (Patil et al., 2019), the selective removal of p16^INK4A^*-*positive senescent cells by the drug ganciclovir reduces the levels of the inflammatory cytokine IL-6 and the matrix protease MMP13, which mitigates age-associated intervertebral disc degeneration. However, what is not clear is whether senescent cells induce aging-related pathologies in ways that do not involve SASP? SNS overactivity participates in the modulation of a multitude of chronic diseases and aging-related disorders. This leads to speculation as to whether there is an association between SNS overactivity and cell senescence.

In the present study, we found a marked increase in SFs in naturally aged mouse tissues and human colon adenoma tissues, which is closely relevant to senescent cells accumulating in aging mice tissues and human colon adenoma tissues. Subsequently, we discovered that senescent cells increase the SFs density by recruiting them. We determined that the axon guidance cue netrin, which is secreted by senescent cells, induces the recruitment of SFs. This may be the mechanism that underlies the SNS hyperactivity-induced aging-related diseases. Furthermore, elevated SFs density leads to impaired brain cognitive performance of naturally aged mice and hepatic steatosis in APOE^−/−^ mice, and treatment with propranolol hydrochloride or 6OHDA reverses the SNS hyperactivity that causes these adverse outcomes. Therefore, it is possible to link SNS overactivity to senescent cells *via* the secretion of netrin, which may be the mechanism by which SNS overactivity induces aging-related pathologies.

To the best of our knowledge, netrin1 either binds to the DCC receptor to promote nerve fiber infiltration or to the UNC5H receptor to repress it, which depends on the relative expression levels of these two kinds of receptors. Our data show that DRG-derived neurons express more DCC receptors than UNC5H receptors (data not shown), suggesting that senescent cell -secreted netrin1 predominately interacts with DCC, and promotes the outgrowth of SF towards senescent cells. Further investigation into the differential expression of the DCC and UNC5H receptors of the SFs in aged tissues environment is warranted.

Although we discovered that elevated SFs density impairs the brain cognitive performance in the brain and represses lipid metabolism in the liver, we know little about the underlying mechanisms by which this elevated SFs density contributes to aging-related pathologies *via* adrenergic neurotransmitters. Renz et al. (2018a) revealed that SFs- secreted -norepinephrine upregulates NGF mRNA *via* the phosphorylated-CREB/ERK pathway, which in turn promotes the outgrowth of SF and increases norepinephrine secretion, increasing the risk of pancreatic cancer. In parallel, we treated 2BS fibroblasts with epinephrine (E) or norepinephrine (NE), and carried out expression chip screening together with RT-qPCR analysis. Our data revealed that treatment with E or NE upregulates the SASP profile, and is implicated in several aging-related pathologies *via* an autocrine or paracrine mechanism, which in turn promotes netrin-1 secretion from senescent cells, leading to elevated SF density (Supplementary Figures 4A,B).

This work links cellular senescence to elevated SNA in aged tissues by the bridge of netrin-1, which contributes to SNS hyperactivity-induced aging-related pathologies, such as Cardiovascular and cerebrovascular diseases, OA, fatty liver, and brain cognitive function. Clearing senescent cells or inhibiting SNA is a promising therapeutic strategy for improving SNS hyperactivity-induced aging-related pathologies, such aging-related diseases mentioned above. Senescent cells-secreted netrin-1 can be regarded as an expanded version of the SASP factor.

Apart from secreting classical SASP, cellular senescent cells also can secrete unclassical factors (such as netrin-1) to interact with microenvironmental components (SFs) in aging tissues thereby modulating aging-related disease. As we known, senescent cells are metabolically active and able to secrete a wealth of known and unknown SASP-like factors, which can alter the microenvironment of aged organization, and complicate aging-associated disorders.

However, the present study also has some limitations. First, although this study revealed that EZH2 is negative regulator, positive regulators of netrin-1 have not been characterized, which need further research to identify the upstream positive regulators of netrin-1. Second, the specific mechanism and signal pathway of SFs regulation of aging-related diseases remain largely unknown. Renz et al. (2018a) have revealed that SFs-secreted NE promotes pancreatic cancer by NGF-BDNF/Trk pathways. In parallel, this study showed that SFs may regulate SASP by secreting E or NE, but the signal pathway regulated by SFs-secreted E or NE needs further investigation.

**Figure 8:**
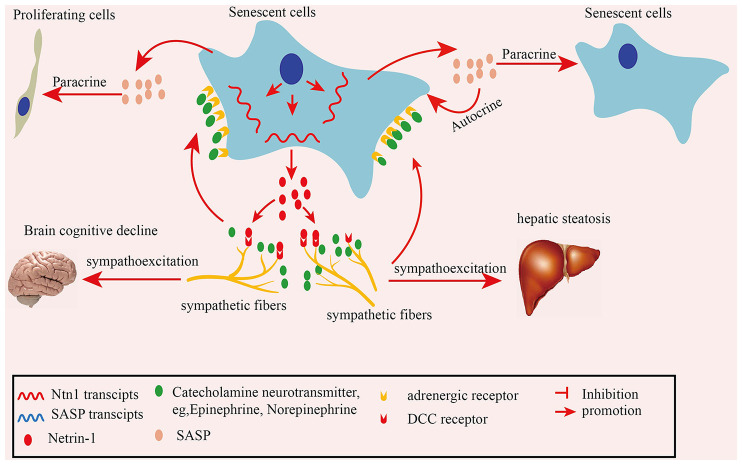
Schematic illustration of how senescent cell-secreted netrin-1 recruits sympathetic fibers (SFs) and modulates aging-related disorders. Senescent cells secreted-netrin-1 induced the and migration and infiltration of SFs, increasing their density in aging tissues. SFs-released transmitter adrenalin upregulated the senescence-associated secretory phenotype (SASP) profile by acting on senescent cells, which accelerated the cellular senescence in an autocrine or paracrine fashion. Increased SFs contributed to cognitive function decline in the brains, and hepatic steatosis in mice, which could be partially alleviated by suppressing SNA.

In summary, in the present study we found elevated SFs densities in naturally aged mouse tissues and human colon adenoma tissues. Mechanistically, senescent cells-secreted netrin-1 induces the infiltration of SFs, which contributes to elevated SF, increasing their density in aging tissues. The recruited SFs upregulate the SASP profile by releasing the adrenalin transmitter, which acts on senescent cells. Furthermore, the increased SF density impairs brain cognitive performance in naturally aged mice and mediates hepatic steatosis in APOE^−/−^ mice (Figure 8). Our finding suggests that clearing senescent cells or targeting netrin-1 are promising preventive strategies for treating SNS hyperactivity-induced aging-related pathologies.

## Data Availability Statement

The datasets analyzed in this study can be found in The Cancer Genome Atlas (https://cancergenome.nih.gov/).

## Author Contributions

ZM, YC and ZW conceived the idea. ZM, AY and XZ discussed the data and wrote the manuscript. AY, JW and XZ designed and performed the experiments and analyzed the data. AY, JW and XZ performed the experiments and collected the data. AY and KC established surgery-induced OA models and administered senolytics into joint cavity. All authors contributed to the article and approved the submitted version.

## Conflict of Interest

The authors declare that the research was conducted in the absence of any commercial or financial relationships that could be construed as a potential conflict of interest.
