# Supplementary material for: Senescent Cell-Secreted Netrin-1 Modulates Aging-Related Disorders by Recruiting Sympathetic Fibers
Source: Front Aging Neurosci. 2020 Dec 16;12:507140. doi: 10.3389/fnagi.2020.507140 (PMC7772213; doi:10.3389/fnagi.2020.507140)
Supplement: Supplementary file 1 [file Table_2.DOCX]

**Senescent cell-secreted netrin-1 modulates ageing-related disorders by recruiting sympathetic fibres**

**Supplementary Figures**


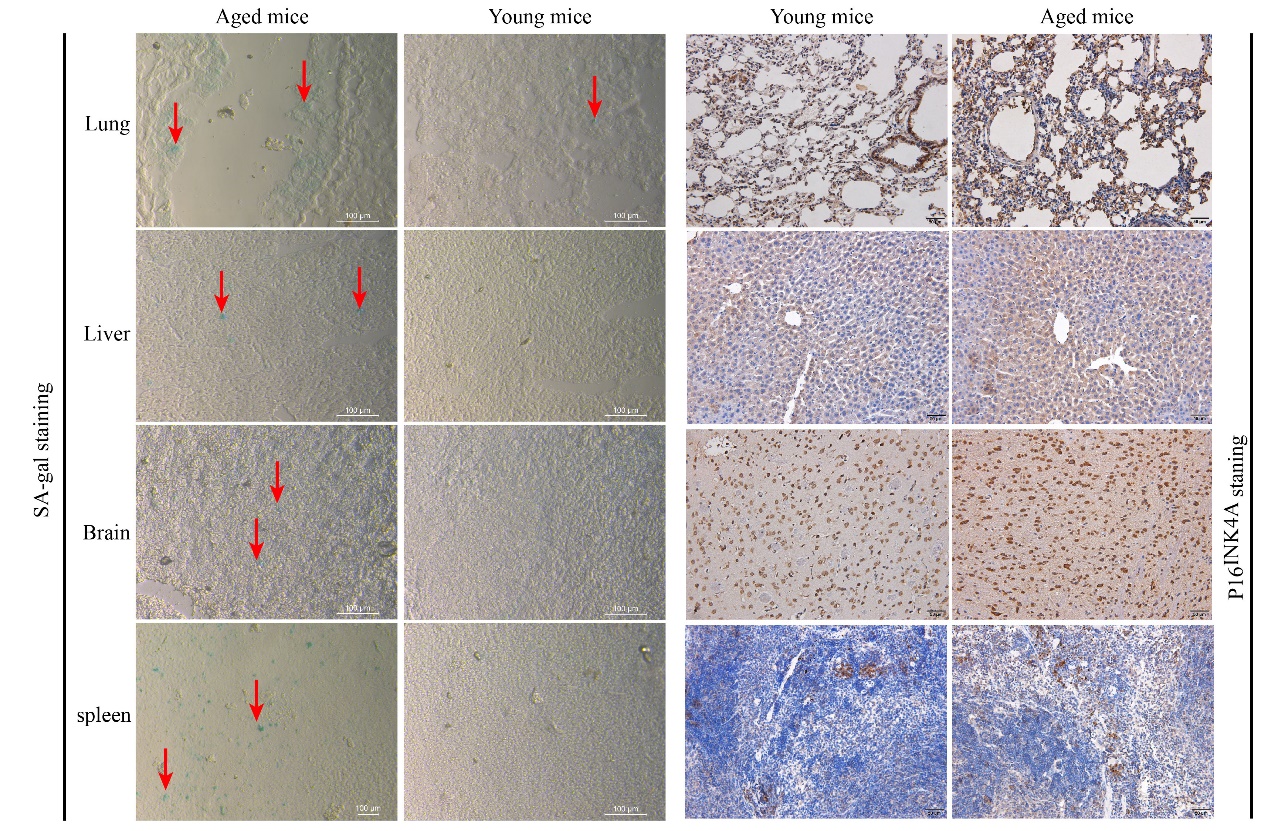


**Supplementary Figure 1 Senescence-associated markers detection of naturally aged mouse tissues and young mouse tissues.** P16^INK4A^ and SA-gal staining was performed to identify naturally aging mouse tissue samples as an aged mouse tissue samples, red arrow indicating positive SA-gal staining, scale bar,100 μm for SA-gal staining and 50 μm for P16^INK4A^.


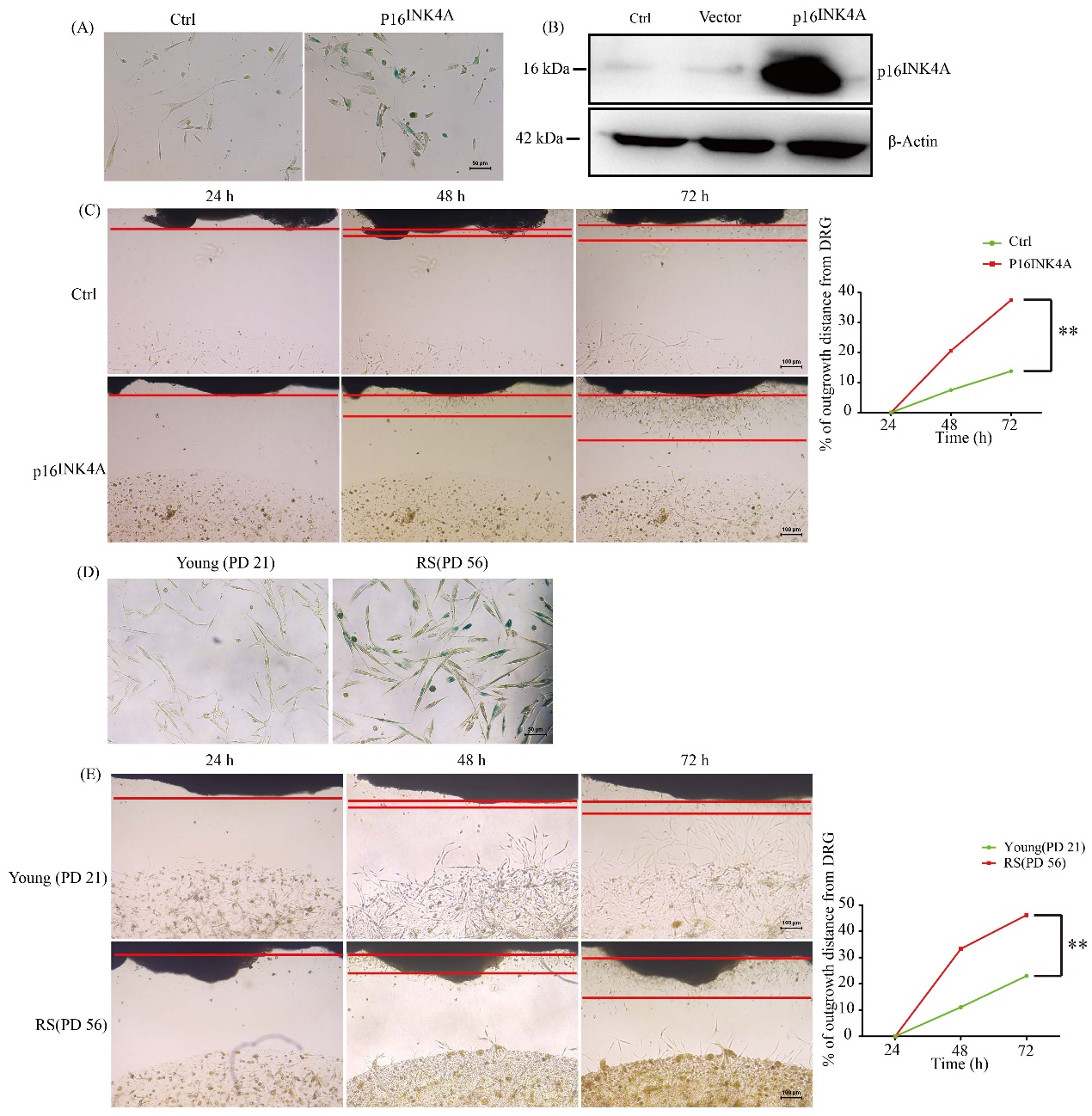


**Supplementary Figure 2** **P16-indued premature senescent 2BS fibroblasts or replicate senescent 2BS fibroblasts promoted sympathetic fibres outgrowth**. （A）SA-gal staining of 2BS fibroblasts infected with vector or P16^INK4A^, scale bar, 50 μm.(B) Western blot analysis determined P16^INK4A^ expression of 2BS fibroblasts infected with or without vector and P16^INK4A^. (C) DRG cocultured with 2BS fibroblasts infected with or without vector and P16^INK4A^, ***P* < 0.01, scale bar, 100 μm. (D) SA-gal staining of young 2BS fibroblasts (PD 21) or replicate senescent 2BS fibroblasts (PD 56), scale bar, 50 μm. (E) DRG cocultured with young 2BS fibroblasts (PD 21) or replicate senescent 2BS fibroblasts (PD 56), ***P* < 0.01, scale bar, 100 μm.


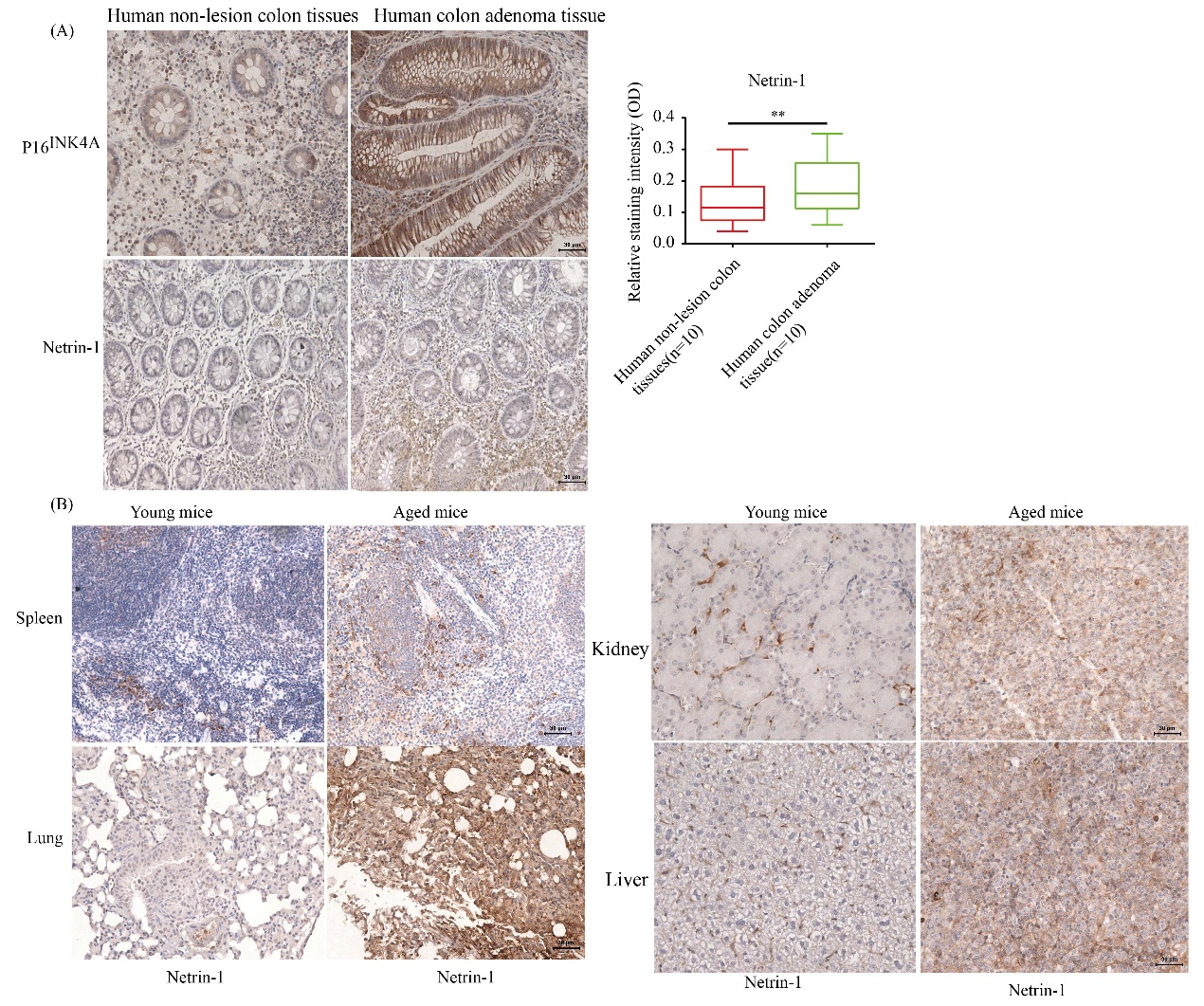


**Supplementary Figure 3.** **netrin-1 expression level is significantly elevated in naturally aged mice tissues and human colon adenoma tissues.** (A and B) IHC analyzed netrin-1 expression level in naturally aged mice tissues, young mice tissues, human non-lesion colon tissues and human colon adenoma tissues, scale bar,30μm ***P* < 0.01,n=10 for A and 3 for B.


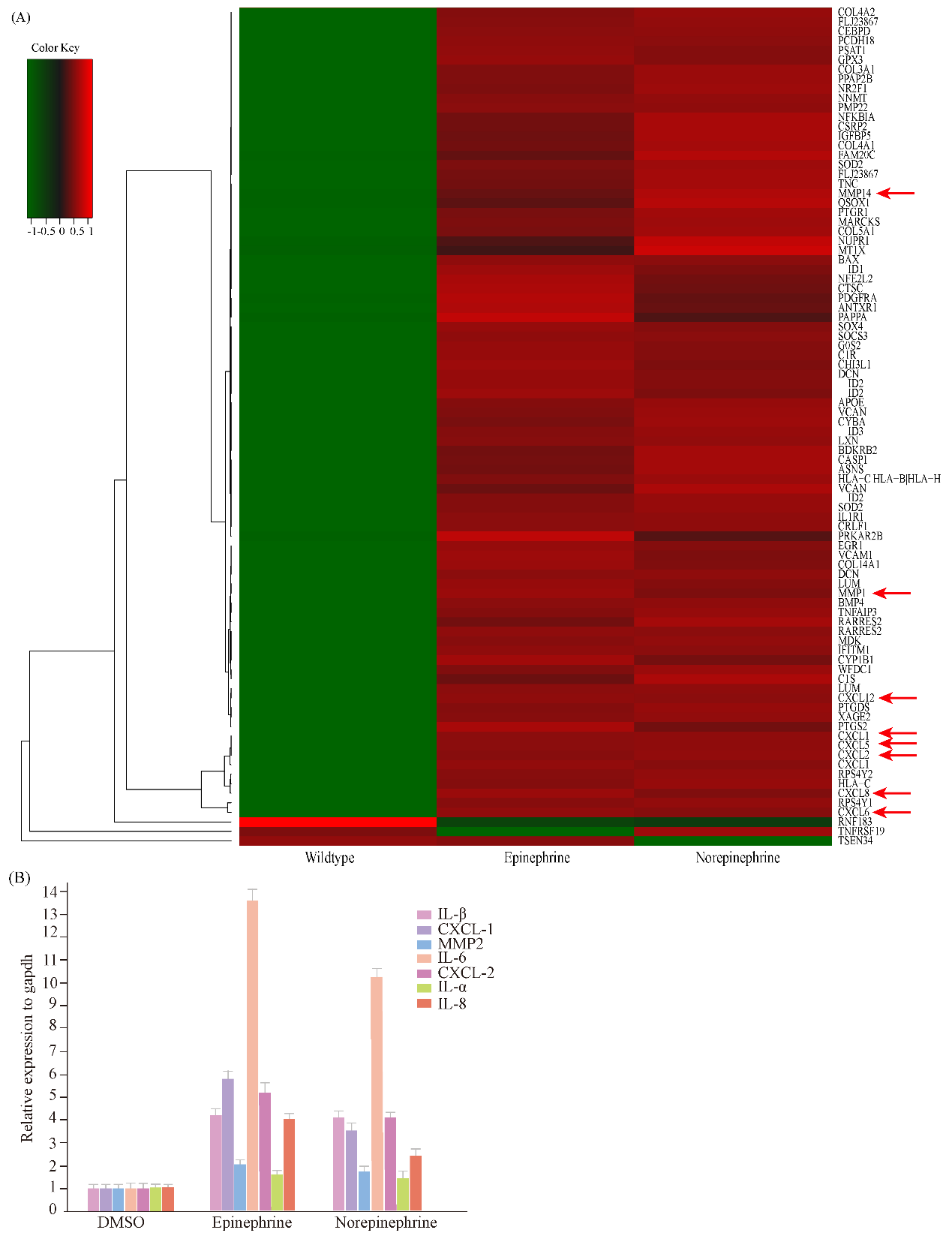


**Supplementary Figure 4.** **Epinephrine or Norepinephrine treatment upregulate SASP profile in young 2BS fibroblasts.** (A and B) Cluster heat maps and RT-qPCR analysis revealed the differentially expressed SASP profile in young 2BS fibroblasts treated with epinephrine or norepinephrine, red arrow denotes the altered SASP components.
